# A revision of brain composition in Onychophora (velvet worms) suggests that the tritocerebrum evolved in arthropods

**DOI:** 10.1186/1471-2148-10-255

**Published:** 2010-08-21

**Authors:** Georg Mayer, Paul M Whitington, Paul Sunnucks, Hans-Joachim Pflüger

**Affiliations:** 1 Institute of Biology II: Animal Evolution & Development, University of Leipzig, Talstrasse 33, D-04103 Leipzig, Germany; 2 Department of Anatomy and Cell Biology, University of Melbourne, Victoria 3010, Australia; 3 School of Biological Sciences and Australian Centre for Biodiversity, Monash University, Melbourne, Victoria 3800, Australia; 4 Institut für Biologie, Neurobiologie, Freie Universität Berlin, Königin-Luise-Str. 28-30, D-14195 Berlin, Germany

## Abstract

**Background:**

The composition of the arthropod head is one of the most contentious issues in animal evolution. In particular, controversy surrounds the homology and innervation of segmental cephalic appendages by the brain. Onychophora (velvet worms) play a crucial role in understanding the evolution of the arthropod brain, because they are close relatives of arthropods and have apparently changed little since the Early Cambrian. However, the segmental origins of their brain neuropils and the number of cephalic appendages innervated by the brain - key issues in clarifying brain composition in the last common ancestor of Onychophora and Arthropoda - remain unclear.

**Results:**

Using immunolabelling and neuronal tracing techniques in the developing and adult onychophoran brain, we found that the major brain neuropils arise from only the anterior-most body segment, and that two pairs of segmental appendages are innervated by the brain. The region of the central nervous system corresponding to the arthropod tritocerebrum is not differentiated as part of the onychophoran brain but instead belongs to the ventral nerve cords.

**Conclusions:**

Our results contradict the assumptions of a tripartite (three-segmented) brain in Onychophora and instead confirm the hypothesis of bipartite (two-segmented) brain composition. They suggest that the last common ancestor of Onychophora and Arthropoda possessed a brain consisting of protocerebrum and deutocerebrum whereas the tritocerebrum evolved in arthropods.

## Background

The head of arthropods is a specialised anterior body region, which is distinguished by fused segments and several pairs of modified appendages [1,2]. These appendages serve for swimming, feeding, defence, or sensory perception, and their movements are coordinated by a complex brain situated within the head. Despite over a century of intense research in this area, the ancestral composition of the arthropod head remains obscure and is one of the most controversial topics in zoology [2-8]. Fossils have contributed much to our knowledge [1,4,8], but their limited preservation constrains definitive conclusions about the degree of cephalisation in the last common ancestor of Panarthropoda (Onychophora + Tardigrada + Arthropoda).

**Figure 1:**
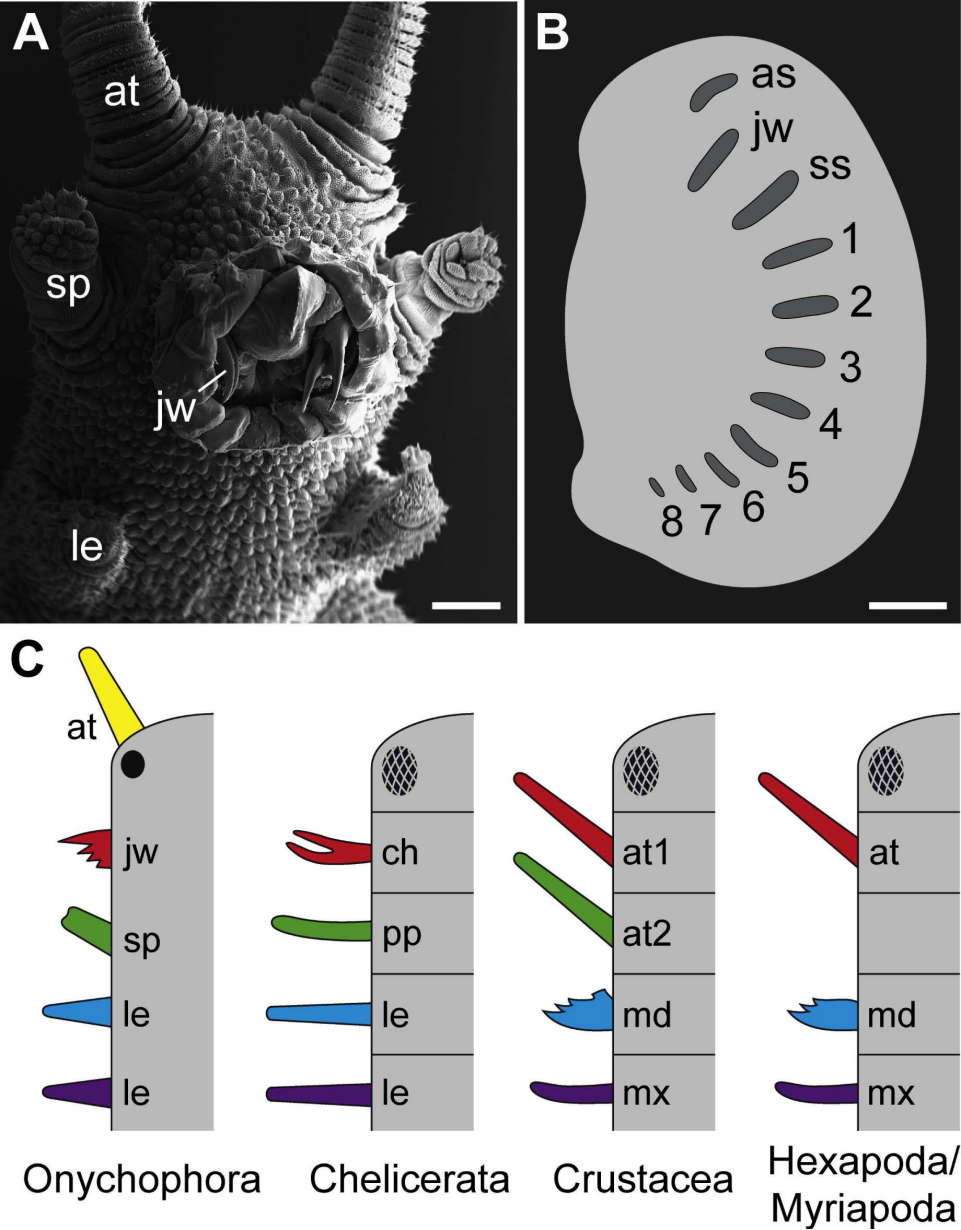
**Head composition and homology of cephalic appendages in Onychophora and Arthropoda**. (A) Ventral view of onychophoran "head" showing three pairs of modified appendages: antennae (at), jaws (jw), and slime papillae (sp). Scanning electron micrograph. Scale bar: 500 μm. (B) Diagram of expression pattern of segment polarity gene *engrailed *in an onychophoran embryo in lateral view (based on fig. 1a from [25]). Scale bar: 200 μm. Note that there are only three anterior expression domains corresponding to posterior borders of antennal (as), jaw (js), and slime papilla segments (ss), in addition to eight trunk segments (numbered). (C) Alignment and serial homology of anterior appendages in Onychophora and the four major arthropod groups [after [2]]. Note that the onychophoran eyes (black filled circle) may be homologous to the median ocelli [62] rather than to the compound eyes of arthropods (checked ovals), although all these ocular structures belong to the same, anterior-most body segment. Abbreviations: as, antennal segment; at, antenna; at1, first antenna; at2, second antenna; ch, chelicera; jw, jaw; js, jaw segment; le, leg; md, mandible; mx, maxilla; pp, pedipalp; sp, slime papilla; ss, slime papilla segment.

The extant Onychophora are a key group when considering this issue, since they are close relatives of arthropods and resemble Cambrian lobopodians [9-13], while their internal anatomy and embryology are accessible for detailed examination. As in various lobopodians, the onychophoran "head" is not clearly delineated from the trunk, but shows three pairs of modified appendages: sensory antennae, jaws situated within the mouth cavity, and slime papillae, which are used for defence and capturing prey organisms (Figure 1A). These modified appendages have been assigned to each body segment by studying embryogenesis, which revealed that the antennae belong to the first (ocular) body segment, the jaws to the second, and the slime papillae to the third segment [14-20]. Most importantly, these studies have provided no evidence of any additional vestigial cephalic segments [21-24] in Onychophora. This is supported by the expression data of segment polarity genes in onychophoran embryos [25], which show only three domains anterior to the leg-bearing segments, corresponding to the three cephalic segments (Figure 1B).

Based on various studies of embryology [14-20], including the expression data of the anterior Hox genes *labial*, *proboscipedia*, *Hox3 *and *Deformed *[26], the onychophoran "head" appendages can therefore be aligned with the corresponding appendages of arthropods (Figure 1C). According to this alignment, the onychophoran antennae are either serial homologues of the arthropod labrum or, alternatively, the corresponding pair of appendages may have been lost in arthropods - an issue that is still controversial [5,27-29]. (It has also been argued that the arthropod labrum is a modified appendage of the third body segment [30]. However, the Hox gene expression data referred to above, together with the common expression of the anterior marker *six3 *in the insect labrum and onychophoran antenna [26], speak against this possibility.) Since the onychophoran antennae belong to the anterior-most body segment bearing the eyes [19,20], they cannot be homologised with the chelicerae of chelicerates or the (first) antennae of crustaceans, insects, and myriapods, which belong to the second body segment [2,3,31]. The chelicerae and the (first) antennae of arthropods are instead serially homologous to the onychophoran jaws (Figure 1C). The onychophoran slime papillae are, in turn, serially homologous to the pedipalps of chelicerates and to the second antennae of crustaceans whereas the corresponding pair of appendages was lost in hexapods and myriapods [review [2]].

**Figure 2:**
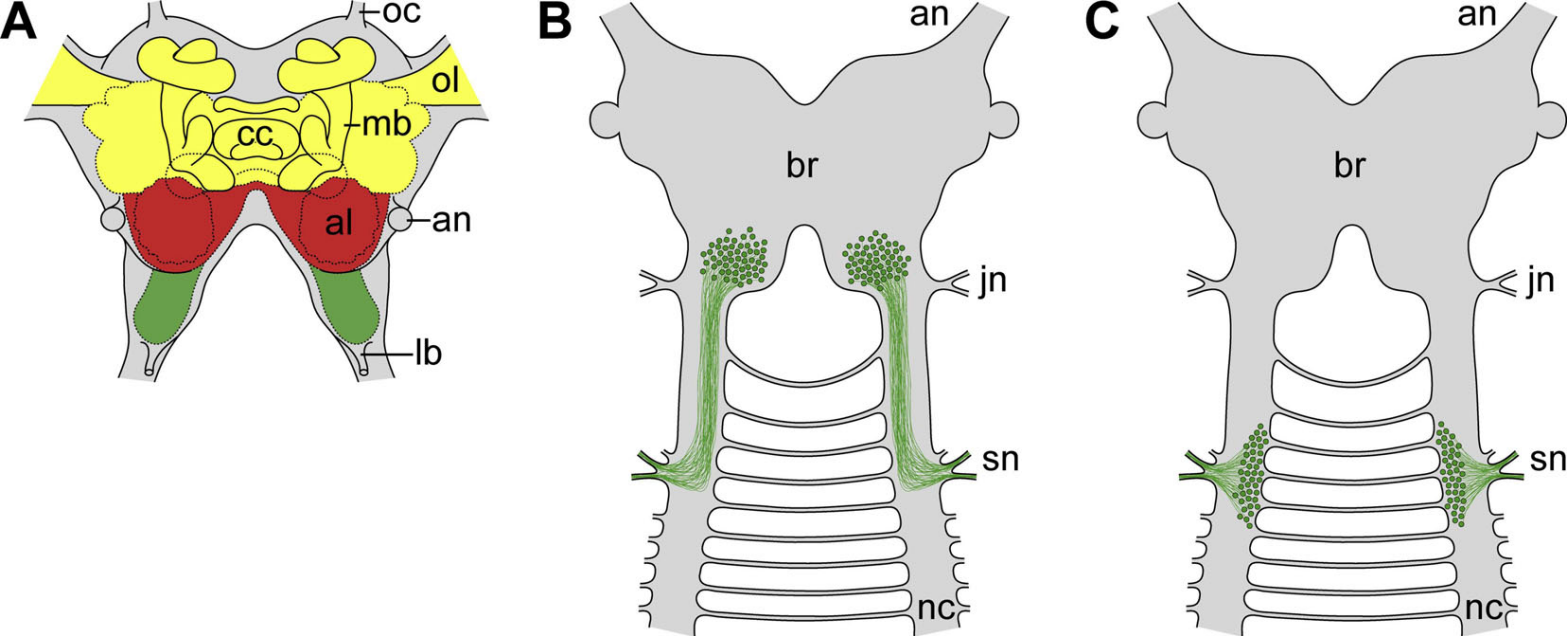
**Subdivision of arthropod brain and alternative possibilities for the position of neuronal cell bodies innervating the third pair of cephalic appendages in Onychophora**. (A) Position of protocerebral (yellow), deutocerebral (red) and tritocerebral structures (green) in the brain of the fruitfly *Drosophila melanogaster *[modified from [32]]. (B) A position within the brain of neuronal cell bodies innervating the onychophoran slime papillae, as shown in this diagram, would support the existence of a region of the onychophoran brain equivalent to the arthropod tritocerebrum. (C) A position outside the brain of neuronal cell bodies innervating the onychophoran slime papillae, as shown in this diagram, would speak against the existence of a tritocerebrum in the onychophoran brain. Abbreviations: al, antennal lobe; an, antennal nerve; br, cerebral ganglion or brain; cc, central complex; jn, jaw nerve; lb, labral nerve; mb, mushroom body; nc, nerve cord; oc, ocellar nerve; ol, optic lobe; sn, slime papilla nerves.

This alignment of head segments is reflected in the organisation of the central nervous system. Three major brain regions are generally recognised in arthropods (Figure 2A): the protocerebrum (forebrain), the deutocerebrum (midbrain), and the tritocerebrum (hindbrain), corresponding to the three anterior-most body segments [2,27,31-35]. Such an organisation has also been suggested for the Onychophora, based on studies of adult brain anatomy and its neuropilar structure [21,22,31]. However, an alternative view [36,37] suggests that the onychophoran brain or "cerebral ganglion" [38,39] is bipartite and does not include the region homologous to the arthropod tritocerebrum.

One feature that has previously been used to determine the segmental organisation of the brain in Onychophora is the position and number of transverse neuropils in the adult [31]. Three major neuropils have been identified, leading to the conclusion that the onychophoran brain is tripartite. However, this rests on the assumption that each neuropil arises from a separate segment during development - an issue, which has not been clarified thus far. An additional feature that could be used to identify the degree of segmentation of the onychophoran brain is the position of neuronal cell bodies innervating the head appendages. If the cell bodies of neurons innervating the tritocerebrum were found to lie within the brain (Figure 2B), the hypothesis of tripartite organisation [31] would be supported. In contrast, a position of these neuronal cell bodies found outside the brain (Figure 2C) would speak against the existence of the tritocerebrum in Onychophora.

To clarify the segmental composition of the onychophoran brain, we combined two approaches. First, we studied brain development to determine the embryonic origin of transverse neuropils. Second, we analysed the position of neuronal cell bodies innervating the cephalic appendages. Our results show that the major transverse neuropils of the onychophoran brain arise from only one (the anterior-most) body segment, and that only the antennae and jaws are innervated by the brain. These findings suggest that the onychophorans show a lower degree of cephalisation in relation to their brain organisation than the arthropods and that the tritocerebrum was not integrated into the brain in the last common ancestor of Onychophora and Arthropoda.

## Results and Discussion

### The formation of onychophoran brain neuropils involves only one segment

Despite two recent and extensive studies of brain development in Onychophora [19,40], the embryonic origin and segmental identities of transverse brain neuropils, other than the first ("antennal") commissure, remain unclear. Strausfeld et al. [31] subdivided the adult onychophoran brain into protocerebrum, deutocerebrum and tritocerebrum by analysing series of histological and silver- and osmium-stained sections and assessing the number and spatial separation of brain neuropils. To clarify whether these brain neuropils have independent origins from different segments, we examined brain development in onychophoran embryos using an antibody raised against acetylated α-tubulin. This antibody labels mainly nerve tracts and neuropils in the developing nervous system [19,40-42].

**Figure 3:**
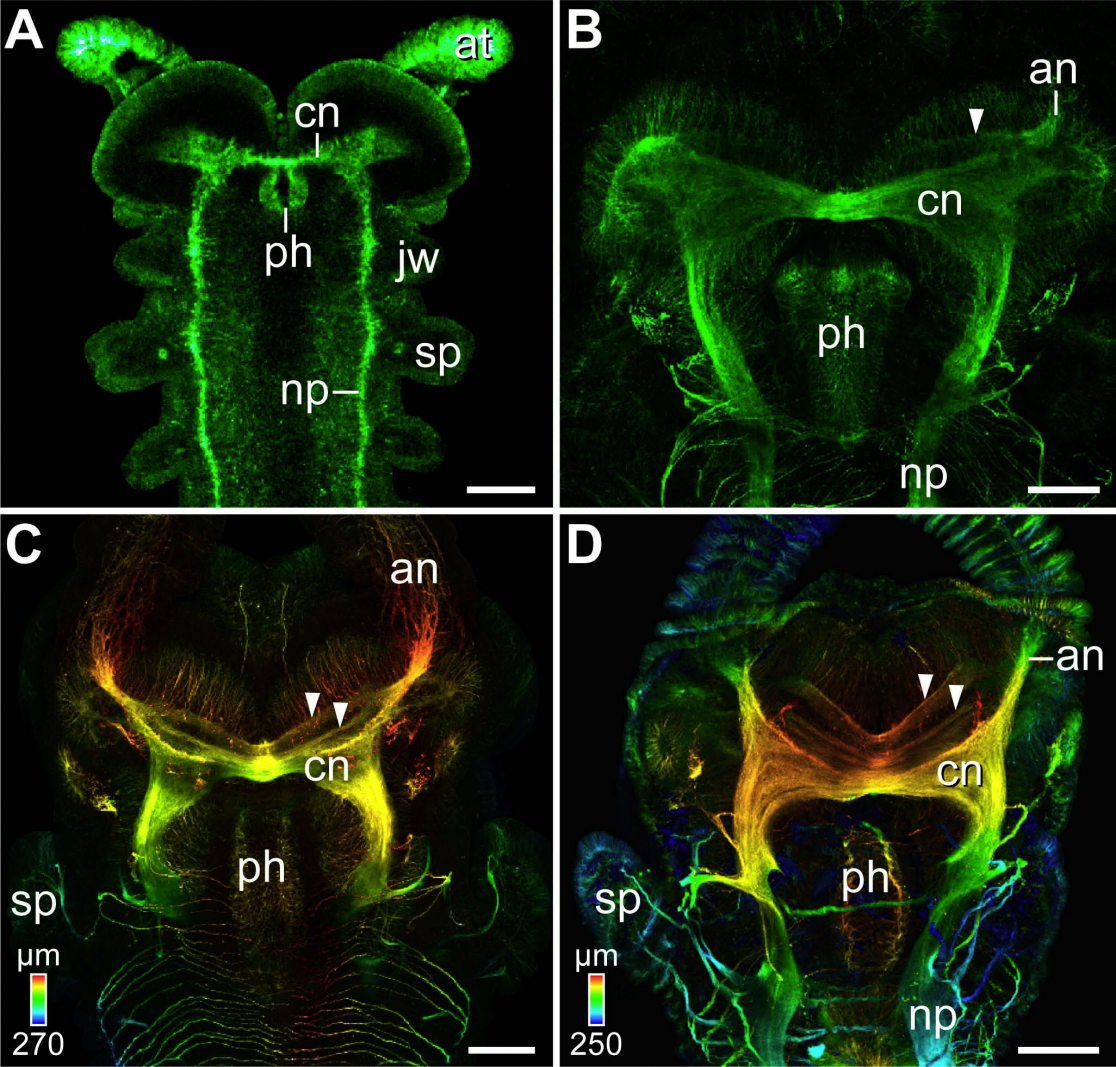
**Development of major neuropils in the onychophoran brain**. Heads of embryos at progressively older developmental stages in dorsal view. Anti-acetylated α-tubulin immunolabelling. Confocal maximum projections (A, B) and depth-coded projections (C, D). Anterior is up. (A) Central neuropil (cn) arises from a single transverse commissure in the antennal segment in an early *Euperipatoides rowelli *embryo. Scale bar: 200 μm. (B-D) Further stages of brain development in embryos of *Epiperipatus isthmicola*. Scale bars: 100 μm. A second transverse neuropil (arrowhead in B) and a third neuropil (arrowheads in C and D) arise anterior to the original commissure. Abbreviations: an, antennal nerve; at, antenna; cn, developing central brain neuropil; jw, jaw anlage; np, future nerve cord neuropil; ph, pharynx; sp, slime papilla.

At an early stage, we detected only one transverse commissure in the anterior-most body segment (Figure 3A - aligning this figure with the regions of *engrailed *expression shown in figs. 1d and f in [25] confirms our assignment of segmental identity). During development, this commissure forms the central neuropil, which subsequently gives rise to a second and a third neuropil (Figure 3B-D). No other transverse neuropils appear posterior to the central neuropil later in development [see also [19,40]]. Thus, the three neuropils identified as proto-, deuto- and tritocerebrum in a previous study [31] do not arise from three different segments. We suggest therefore that the position and physical separation of neuropils in the adult brain alone [22,31] is an unreliable criterion for identifying its segmental organisation. Thus, our immunolabelling experiments do not resolve the controversy of bipartite [36,37] versus tripartite [21,22,31] brain composition in Onychophora. Alternative approaches are required to decide between these two hypotheses.

### Retrograde axonal tracing reveals that the tritocerebrum is absent from the onychophoran brain

The position of neurons that project out the segmental nerves within the onychophoran head might be a key feature for determining the segmental identity of different brain regions. We therefore performed retrograde axonal tracing studies (backfills) of segmental cephalic nerves in adult onychophorans, using dextran coupled to different fluorochromes as a tracer [43].

**Figure 4:**
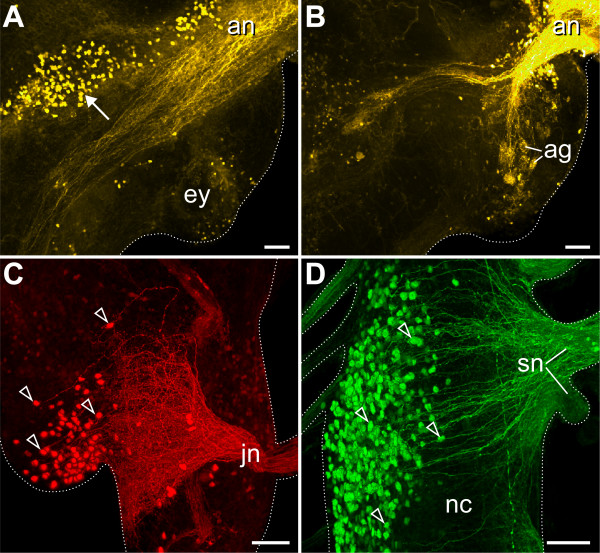
**Position of neuronal cell bodies innervating segmental cephalic appendages in the onychophoran *Euperipatoides rowelli***. Differential staining by retrograde fills with dextran. Confocal projections. Scale bars: 50 μm. (A) Antennal nerve (an) filled with dextran-tetramethylrhodamine. Note the antero-median position of neuronal cell bodies (arrow) within the protocerebral brain region. Anterior is in the upper right corner. (B) Partial projection of the same stack as in A showing that some filled axons of the antennal nerve terminate in the antennal glomeruli (ag). (C) Detail of a jaw nerve (jn) filled with dextran-tetramethylrhodamine. (D) Detail of slime papilla nerves (sn) filled with dextran-fluorescein. Arrowheads indicate the position of neuronal cell bodies in C and D. Abbreviations: ag, antennal glomeruli; an, antennal nerve; ey, eye; jn, jaw nerve; nc, nerve cord; sn, slime papilla nerves.

**Figure 5:**
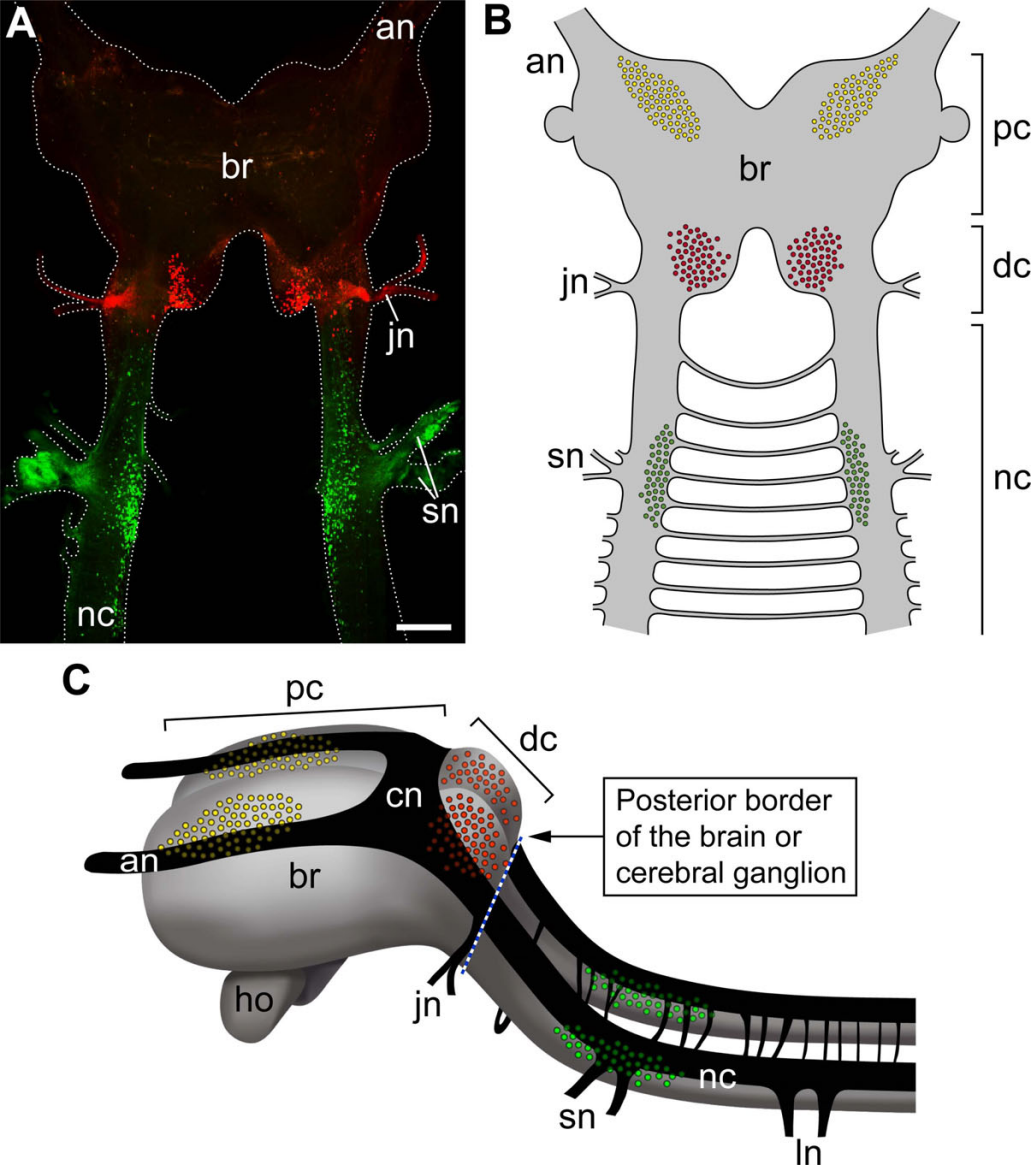
**Position of neuronal cell bodies innervating segmental cephalic appendages in Onychophora and posterior border of the onychophoran brain**. (A) Overview of the differential staining of segmental cephalic nerves in *Euperipatoides rowelli *by retrograde fills with dextran (confocal projection). Jaw nerves (jn) and slime papilla nerves (sn) from both sides of the body were filled with dextran-tetramethylrhodamine (red) and dextran-fluorescein (green). Anterior is up. Scale bar: 200 μm. (B) Diagram summarising the location of neuronal cell bodies innervating the antennae (yellow), jaws (red), and slime papillae (green). Note that the cell bodies of neurons innervating the slime papillae lie outside the brain. (C) Anterior portion of the onychophoran nervous system (reconstruction based on confocal images of an immunolabelled embryo; see Additional file 1, Figure S1). The position of neurons innervating the segmental cephalic appendages (colour coding as in B) is mapped on the reconstructed nervous system. Blue dashed line indicates the posterior brain border behind the cerebral accumulation of neurons. Abbreviations: an, antennal nerve; br, cerebral ganglion or brain; cn, central neuropil; dc, deutocerebrum; ho, hypocerebral organ; jn, jaw nerve; ln, leg nerves; nc, nerve cord; pc, protocerebrum; sn, slime papilla nerves.

We found that the cell bodies of neurons innervating the antennae lie within the brain (Figure 4A), in the region corresponding to the arthropod protocerebrum [19,20,31,44]. Some of the filled axons of the antennal nerve terminate in glomerular structures (Figure 4B), which have been described previously [31,38,44]. The cell bodies innervating the jaws and the slime papillae lie adjacent to the base of their corresponding nerves: the jaw neurons are situated in the posterior-most (deutocerebral) region of the cerebral ganglion whereas those innervating the slime papillae lie in a more postero-ventral position within the nerve cord (Figures 4C, D and 5A-C).

Our data show that the neurons innervating the slime papillae are located within the ventral nerve cord and, thus, outside the brain, the posterior border of which lies just posterior to the jaw nerves and anterior to the slime papillae nerves (Figure 5C). This placement of the posterior brain border is consistent with all previous studies of the adult onychophoran brain anatomy [e.g. [21,22,31,36-39,44,45] and references therein]. The neurons innervating the slime papillae cannot be considered part of the brain as the corresponding region of the central nervous system does not show any particular condensation of neurons or other morphological characteristics that would distinguish it from the medullary nerve cords.

**Figure 6:**
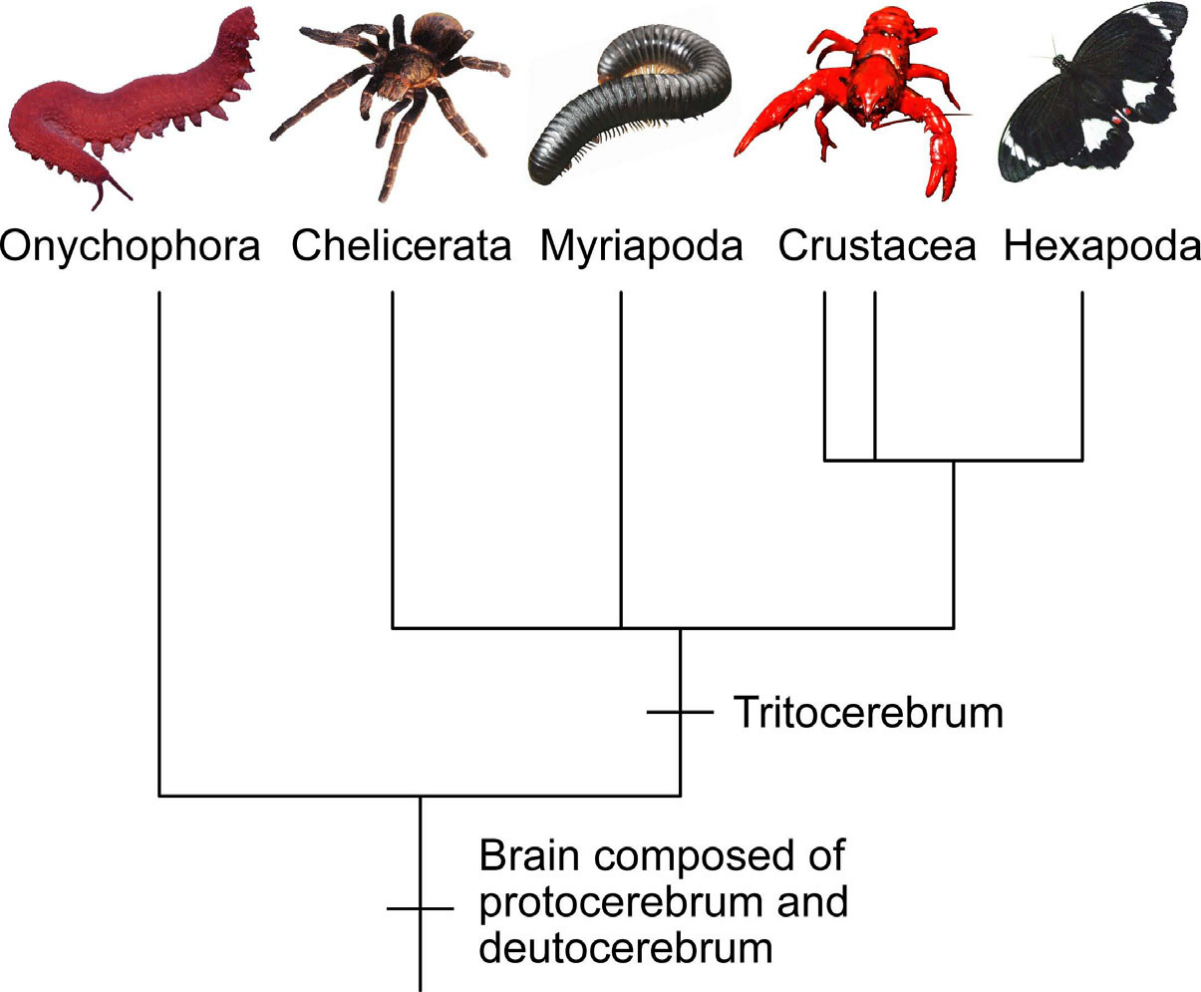
**Implications of the current findings for the evolution of the arthropod brain**. The brain of the last common ancestor of Onychophora and Arthropoda was composed of the protocerebrum and deutocerebrum whereas the tritocerebrum, as part of the brain, evolved in arthropods (chelicerates, myriapods, crustaceans, and hexapods). The phylogenetic position of Myriapoda is unresolved [42,63]. Double lines for Crustacea indicate that this group might not be monophyletic [64].

Our backfill data reveal that only the cell bodies of neurons supplying the antennae and jaws lie within the brain whereas the region corresponding to the arthropod tritocerebrum belongs to the nerve cord (Figure 5A-C). This finding contradicts previous assumptions of a tripartite (three-segmented) brain in Onychophora [2,21,22,24,31]. The absence of the tritocerebrum from the onychophoran brain implies that the bipartite brain composition is an ancestral feature of Onychophora. An alternative scenario proposing that the tritocerebrum might have become separated from the onychophoran brain secondarily is unlikely since one would have to assume opposite relocation events during the evolution of the onychophoran head: while the slime papillae have been incorporated into the head by moving anteriorly, the corresponding brain region would have become separated from the cerebral ganglion by a postero-ventral relocation. Moreover, this region would have lost its ganglionic organisation and reverted back to a portion of the medullary nerve cord. Studies of early neural development in the onychophoran embryo [19,41,42] have revealed no evidence for an origin in the presumptive brain of the neural precursors that give rise to neurons innervating the slime papillae. We therefore regard this scenario as unlikely and suggest that the tritocerebrum was not present in the last common ancestor of Onychophora and Arthropoda but rather evolved in arthropods (Figure 6).

## Conclusions

In summary, our findings suggest an increase in the number of segmental brain regions in the (pan)arthropod lineage, from two in the last common ancestor of Onychophora and Arthropoda, to at least three in various arthropods [e.g. [2,27,31,32]]. This evolutionary sequence may help clarify the phylogenetic position of Tardigrada (water bears), which is still controversial. Currently, tardigrades are regarded as either the sister group of arthropods, of onychophorans, of onychophorans plus arthropods, or of one of the cycloneuralian taxa (nematodes, kinorhynchs, and allies) [10,11,41,46-54]. Our findings suggest that the number of segments in the tardigrade brain, which remains unclear [48,55-58], will be a key feature in elucidating the position of this animal group within the Ecdysozoa.

Furthermore, our suggestion of a two-segmented brain in the last common ancestor of Onychophora and Arthropoda challenges the hypothesis that a tripartite brain existed in the last common ancestor of the bilaterally symmetrical animals, the so-called "urbilaterian" [59,60]. Such a brain is absent in all protostomes apart from arthropods. Moreover, the closest relatives of chordates, including hemichordates and echinoderms [48], lack a centralised brain. We therefore suggest that similar gene expression patterns in the anterior body region of arthropods and vertebrates [59,60] are not related to brain segmentation but rather to a general patterning of the antero-posterior body axis in these animals.

## Methods

Specimens of *Euperipatoides rowelli *Reid, 1996 and *Epiperipatus isthmicola *(Bouvier, 1902) were collected and handled and the embryos staged and labelled with an antibody raised against acetylated α-tubulin as described previously [41,42]. For neuronal tracing, adult brain nerves were dissected in physiological saline based on onychophoran blood composition [61]. Retrograde fills of the antennal nerves (n = 3), jaw nerves (n = 9), and slime papillae nerves (n = 7) were carried out with dextran (MW 3000) coupled to either tetramethylrhodamine or fluorescein according to standard procedures used for arthropods [43]. Scanning electron microscopy and immunohistochemistry were performed as described previously [42]. Stained specimens were dehydrated through a methanol series and mounted between two cover slips in a 2:1 mixture of benzyl benzoate and benzyl alcohol. Confocal laser-scanning microscopy and image processing were carried out as described previously [41,42].

## Authors' contributions

GM conceived, designed and performed the experiments and wrote the first draft of the manuscript. PS and PMW helped with specimen collection. PMW and H-JP contributed laboratory space, reagents, materials and analysis tools. All authors participated in the discussion of the results and the preparation of the final manuscript.

## Supplementary Material

Additional file 1 **Figure S1**. Anterior nervous system in an almost fully developed embryo of the onychophoran *Epiperipatus isthmicola*. Confocal maximum projection. Dorso-lateral view (anterior is left, dorsal is up). Anti-acetylated α-tubulin immunolabelling. Abbreviations: an, antennal nerves; br, brain; cn, developing central brain neuropil; jn, jaw nerve; ln, paired leg nerves; mo, mouth position; nc, ventrolateral nerve cords; sn, slime papilla nerves. Scale bar: 200 μm. Click here for file
