# Supplementary material for: A revision of brain composition in Onychophora (velvet worms) suggests that the tritocerebrum evolved in arthropods
Source: BMC Evol Biol. 2010 Aug 21;10:255. doi: 10.1186/1471-2148-10-255 (PMC2933641; doi:10.1186/1471-2148-10-255)
Supplement: Additional file 1 — Figure S1. Anterior nervous system in an almost fully developed embryo of the onychophoran Epiperipatus isthmicola. Confocal maximum projection. Dorso-lateral view (anterior is left, dorsal is up). Anti-acetylated α-tubulin immunolabelling. Abbreviations: an, antennal nerves; br, brain; cn, developing central brain neuropil; jn, jaw nerve; ln, paired leg nerves; mo, mouth position; nc, ventrolateral nerve cords; sn, slime papilla nerves. Scale bar: 200 μm. [file 1471-2148-10-255-S1.PDF]

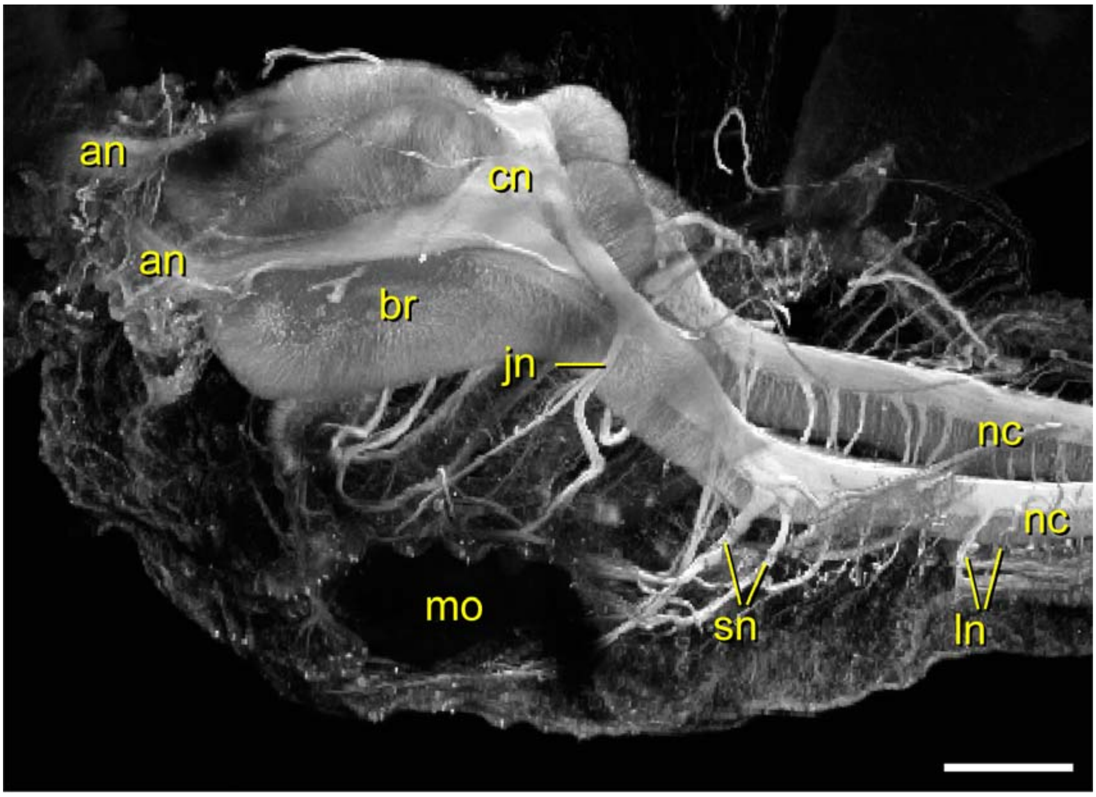

Figure S1

Anterior nervous system in an almost fully developed embryo of the onychophoran *Epiperipatus isthmicola*. Confocal maximum projection. Dorso-lateral view (anterior is left, dorsal is up). Anti-acetylated α-tubulin immunolabelling. Abbreviations: an, antennal nerves; br, brain; cn, developing central brain neuropil; jn, jaw nerve; ln, paired leg nerves; mo, mouth position; nc, ventrolateral nerve cords; sn, slime papilla nerves. Scale bar: 200 μm.
